# Transient receptor potential vanilloid type 4 (TRPV4) promotes tumorigenesis *via* NFAT4 activation in nasopharyngeal carcinoma

**DOI:** 10.3389/fmolb.2022.1064366

**Published:** 2022-12-22

**Authors:** Peng Zhang, Ke Li, Zhen Wang, Yongjin Wu, Hua Zhang, Fang Ma, Xiao-Yu Liu, Michael C.F. Tong, Xiaochen Ru, Xiangmin Zhang, Xianhai Zeng

**Affiliations:** ^1^ Longgang Otorhinolaryngology hospital and Shenzhen Key Laboratory of Otorhinolaryngology, Shenzhen Institute of Otorhinolaryngology, Shenzhen, Guangdong, China; ^2^ School of Medicine, Southern University of Science and Technology and Shenzhen Middle School, Shenzhen, Guangdong, China; ^3^ Department of Otorhinolaryngology, Head and Neck Surgery, The Chinese University of Hong Kong, Hong Kong, Hong Kong SAR, China; ^4^ School of Medicine and Nursing, Huzhou University, Huzhou, China

**Keywords:** nasopharyngeal carcinoma, TRPV4, tumorigenesis, NFAT4, calcium

## Abstract

Transient receptor potential vanilloid type 4 (TRPV4) can function as an oncogene or tumor suppressor depending on the tumor types. However, little is known regarding the effect of TRPV4 in nasopharyngeal carcinoma (NPC), a highly prevalent malignancy in Southern China and Southeast Asia. We found that TRPV4 mRNA and protein levels were significantly upregulated in NPC tissues. In addition, activation of TRPV4 in NPC cell lines using GSK1016790A (100 nM) induced a Ca^2+^ influx, whereas pharmacological inhibition or gene knockdown of TRPV4 reduced the proliferation rates of NPC cells. TRPV4 knockdown also decreased the growth of tumor xenografts *in vivo*. Mechanistically, TRPV4-mediated tumorigenesis is dependent on the activation of Ca^2+^/calcineurin/calcineurin-nuclear factor of activated T cell 4 (NFAT4) signaling. Furthermore, NFAT4 protein level was overexpressed in NPC tissues and correlated positively with TRPV4. Taken together, TRPV4 promotes the malignant potential of NPC cells by activating NFAT4 signaling. Our findings highlight TRPV4-NFAT4 axis as a potential therapeutic target in NPC.

## Introduction

Nasopharyngeal carcinoma (NPC) is a malignancy of the head and neck that originates from the nasopharyngeal epithelial cells. It is particularly prevalent in Southern China and Southeast Asia (Chen et al., 2019; Lee H. M et al., 2019). The risk factors include Epstein–Barr virus (EBV) infection, cigarette smoking, genetic susceptibility, and diet (Hildesheim and Wang 2012). The majority of NPC patients are diagnosed in the advanced stage of the disease, and have poor prognosis (Su et al., 2022). Therefore, it is necessary to explore the underlying pathological mechanisms of NPC development and progression in order to identify novel biomarkers.

Transient receptor potential vanilloid type 4 (TRPV4) is a Ca^2+^-permeable non-selective cation channel (Liedtke et al., 2000; Strotmann et al., 2000; Guler et al., 2002; Watanabe et al., 2002; Nilius and Voets 2013). Temperature, osmotic pressure, mechanical force, pH, UVB, lysophosphatidylcholine, lysophosphatidic acid, epoxyeicosatrienoic acids can activate TRPV4 channel (Liedtke et al., 2000; Strotmann et al., 2000; Moore et al., 2013; Chen et al., 2014; Corn, Windham, and Rafat 2020; Chen et al., 2021). It is ubiquitous across different cell types including nerve cells, endothelial cells, epithelial cells and fibroblasts (Garcia-Elias et al., 2014; Rahaman et al., 2014).

The expression of TRP channels are altered depending on the nature of the cancers and play key role in cancer cell proliferation and apoptosis (Bodding 2007). For example, TRPV6 is upregulated in colon cancer, breast cancer, prostate cancer, thyroid cancer and ovary cancer (Santoni et al., 2011). TRPV1 expression is enhanced in colon cancer, pancreatic cancer, bladder cancer, and prostate cancer (Santoni et al., 2011). TRPV4 was elevated in colon cancer, basal-like breast cancer and tumor-derived endothelial cells, but was downregulated in keratinocytes in non-melanoma skin cancer (Nilius et al., 2007; Prevarskaya et al., 2007; Fiorio Pla et al., 2012; Xie et al., 2017; Zhang et al., 2018; Liu et al., 2021). In a recent study, we demonstrated that knocking down TRPV4 in colon cancer cells suppressed their malignant potential by activating PTEN, and decreased their invasiveness *via* inhibition of ZEB1 signaling (Liu et al., 2019; Zhang et al., 2021). On the other hand, activation of TRPV4 enhanced epithelial to mesenchymal transition (EMT) in breast cancer cells, which is critical for metastasis and therapeutic resistance (Azimi et al., 2020). Extracellular fluid viscosity promotes breast cancer cell migration by TRPV4-dependent Hippo pathway (Bera et al., 2022). Furthermore, the TRPV4-mediated Ca^2+^ pathway promotes the proliferation, migration and invasion of gastric cancer cells (Xie et al., 2017). TRPV4 links calcium signaling to DDX3X activity, which is essential for cancer-associated EBV infections (Donate-Macian et al., 2018; Okuno et al., 2019). However, the role of TRPV4 in the progression of NPC has not been examined, and the specific mechanism remains elusive.

Nuclear factor of activated T cells (NFAT) acts as a bridge between Ca^2+^ signaling pathway and target gene expression, and is closely related to the occurrence and development of tumors (Qin et al., 2014). Intestinal epithelial calcineurin/NFAT signaling drives the survival and proliferation of colorectal cancer (CRC) stem cells and promotes cancer development (Peuker et al., 2016). NFAT3 is also highly expressed in skin cancer cells and plays an oncogenic role (Xiao et al., 2017). We previously showed that hypoxia-mediated NFAT4 activation enhances tumor cell invasion and angiogenesis in breast cancer (Liu et al., 2018b). The Ca^2+^ influx through TRPV4 is known to activate NFAT4 in airway smooth muscle cells (ASMCs) (Zhao et al., 2014), although it is unclear whether this signaling contributes to NPC progression.

In this study, we found that TRPV4 protein levels were significantly higher in the NPC tissues resected from patients, as well as in the human NPC cell lines. Downregulation or pharmacological inhibition of TRPV4 significantly inhibited the proliferation of NPC cells *in vitro* and *in vivo*. Taken together, TRPV4 likely exerts its oncogenic functions in NPC through a Ca^2+^-dependent calcineurin/NFAT4 axis. Our findings provide novel insights into the role of TRPV4 in the progression of NPC, and highlights its potential as a therapeutic target.

## Materials and methods

### Drugs and antibodies

GSK2193874 (HY-100720), HC067047 (HY-100208), FK506 (HY-13756) and cyclosporin A (HY-B0579) were purchased from MedChemExpress. The anti-TRPV4 antibody (ACC-034) was from Alomone Labs, the anti-NFAT4 (sc-8405) and anti-ATCB (sc-47778) antibodies were from Santa Cruz Biotechnology, and anti-TATA binding protein (TBP) antibody (ab51841) was obtained from Abcam.

### Ethics statement

The experiments involving clinical samples were approved by the Medical Ethics Committees of Longgang Otorhinolaryngology Hospital, and the study conformed to the principles outlined in the World Medical Association Declaration of Helsinki. Informed consent was obtained from all participants and the specimens were anonymous. This study contained 183 specimens, including 13 NPC tissues and paired adjacent normal fresh-frozen tissues, 107 paraffin-embedded NPC tissues and 63 paraffin-embedded nasopharynx tissues.

### Cell culture

The human NPC cell lines 5–8F, 6–10B and C666-1 were obtained from BeNa Culture Collection, Jiangsu, China. CNE2 and HONE1 were obtained from College of Pharmacy, Guilin Medical University, Guangxi, China. CNE1 was obtained from School of Basic Medical Sciences, Guangzhou University of Chinese Medicine, Guangzhou, China. Cells were maintained in DMEM or RPMI 1640 medium supplemented with 10% fetal bovine serum (FBS), 100 U/ml penicillin, and 100 μg/ml streptomycin. The cells were cultured at 37°C, in a humidified incubator with 95% O_2_ and 5% CO_2_ (Cheng et al., 2019; Liang et al., 2019).

### RT-qPCR

Total RNA was isolated from the cultured cells using the miRNeasy Mini Kit (Qiagen, 74104) according to the manufacturer’s instructions, and reverse transcribed using the RT Master Mix for qPCR kit (MCE, HY-K0511). Finally, qPCR was performed using SYBR Green qPCR Master Mix (MCE, HY-K0522) on the Applied Biosystems 7500 FAST Real Time PCR system. The primer sequences were as follows: Actin forward 5′-CAC​CAT​TGG​CAA​TGA​GCG GTTC-3′, reverse 5′-AGG​TCT​TTG​CGG​ATG​TC CACGT-3′; TRPV4 forward 5′- TCA​CTC​TCA​CCG​CCT​ACT​ACC​A-3′; reverse 5′- CCC​AGT​GAA​GAG​CGT​AAT​GA C-3′;

### Western blotting

Western blotting was performed as previously described (Liu et al., 2019). Total proteins were extracted from the cultured cells using RIPA lysis buffer (Beyotime Biotech). The nuclear fractions were prepared using Nuclear and Cytoplasmic Protein Extraction kit (Beyotime Biotech). The blots were incubated overnight with primary antibodies at 4°C, followed by horseradish peroxidase-conjugated secondary antibodies (Cell Signaling) at room temperature for 2 h. The protein bands were detected using the Pierce™ ECL Western Blotting Substrate (Thermo Scientific).

### Intracellular Ca^2+^ measurement

Briefly, cells were loaded with 0.02% pluronic F-127 (Invitrogen) and 10 μM Fluo-4/AM (Invitrogen) in the dark at 37°C for 30 min. In a GSK1016790A-stimulated Ca^2+^ influx experiments, the change of intracellular Ca2+ in response GSK1016790A (100 nM) was measured by Fluo-4 fluorescence using excitation at 488 nm. Fluorescence of the cells was recorded by a Zeiss LSM880 confocal microscope and the signals relative to the starting signal (F1/F0) were calculated to quantify the cytosolic Ca^2+^ change (Zhang et al., 2016; Xie et al., 2022).

### Cell viability assay

The cells were seeded in 96 well plates, cultured for 24–72 h and incubated with 10 μl/well Cell Counting Kit-8 reagent (MedChemExpress HK-K0301) for 2 h at 37°C. The absorbance was measured at 450 nm using a multi-mode plate reader (Molecular Devices).

### Colony formation assay

The suitably transfected cells were seeded into six well plates and cultured for 7–9 days. Colonies were fixed with 4% PFA and stained with crystal violet (Beyotime Biotech), and those with 50 or more cells were counted.

### EdU assay

The cells were seeded on a confocal dish and labelled using the BeyoClick™ EdU Cell Proliferation Kit with Alexa Fluor 488 (Beyotime Biotech) according to the manufacturer’s protocol. Images were captured on a Leica TCS SP5 confocal microscope.

### siRNA transfection

The cells were transfected with siRNAs using DharmaFECT transfection reagent (GE Dharmacon) as previously described. The siRNA sequences targeting TRPV4 were as follows: siTRPV4#1: 5′-AUC​UUG​GUA​ACA​AAC​UUG​G-3′, and siTRPV4#2: ON-TARGET plus SMART pool against human TRPV4 siRNA (GE Dharmacon, CO, United States of America). The pooled siRNAs against human NFAT4 were obtained from Santa Cruz.

### Immunohistochemistry

Immunohistochemical staining was performed as previously described (Liu et al., 2019). The staining intensity and proportion of stained cells in each specimen were evaluated using the German semi-quantitative scoring system. The intensity of staining was scored as 0: none, 1: weak, 2: moderate and 3: strong, and the proportion of stained cells as 0: 0%, 1: 1%–24%, 2:25%–49%, 3: 50%–74% and 4: 75%–100%. The final score was obtained by multiplying both scores, which ranged from 0 to 12.

### Mouse xenograft model

All experiments were approved by the Animal Experimental Ethics Committee of Shenzhen Institute of E.N.T. and Use of Laboratory Animals published by the US National Institute of Health. HONE1 cells or 6–10B cells infected with lentivirus carrying TRPV4-shRNA were subcutaneously injected into the dorsal flank of nude mice. Tumor volumes were estimated using the formula: volume (mm^3^) = (width)^2^ × length/2.

### Statistical analyses

Statistical analysis was performed using two-tailed Student’s t-test between two groups. Differences among three or more groups were used one-way ANOVA. Cell viability and tumor volume were examined by two-way ANOVA. All analyses were performed with the GraphPad Prism software version 5.0. Data were expressed as mean ± standard error of the mean (SEM) of at least three independent experiments. *p*-value <0.05 was considered statistically significant.

## Results

### TRPV4 is upregulated in NPC tissues

**FIGURE 1:**
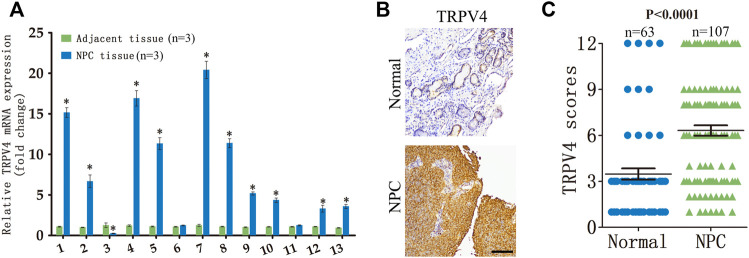
TRPV4 expression in NPC tissues **(A)** Relative TRPV4 mRNA levels in 13 paired tumor and normal nasopharyngeal tissues from NPC patients (n = 3). **(B,C)** Representative images and scores of TRPV4 protein expression in normal nasopharyngeal (n = 63) and NPC tissues (n = 107). Scare bar = 100 μm *p < 0.05. versus NPC tissue.

We analyzed TRPV4 mRNA expression in NPC tissues and paired adjacent normal tissues obtained from 13 patients. TRPV4 was significantly upregulated in 77% (10/13) of the NPC tissues compared to the normal tissues (Figure 1A). Furthermore, immunostaining of NPC (n = 107) and normal nasopharynx tissues (n = 63) showed that the *in situ* expression of TRPV4 protein was markedly higher in the tumors *versus* the normal tissues (Figures 1B, C). Taken together, these data suggested that TRPV4 is aberrantly upregulated in NPC.

### TRPV4 knockdown inhibited the malignant potential of NPC cells

**FIGURE 2:**
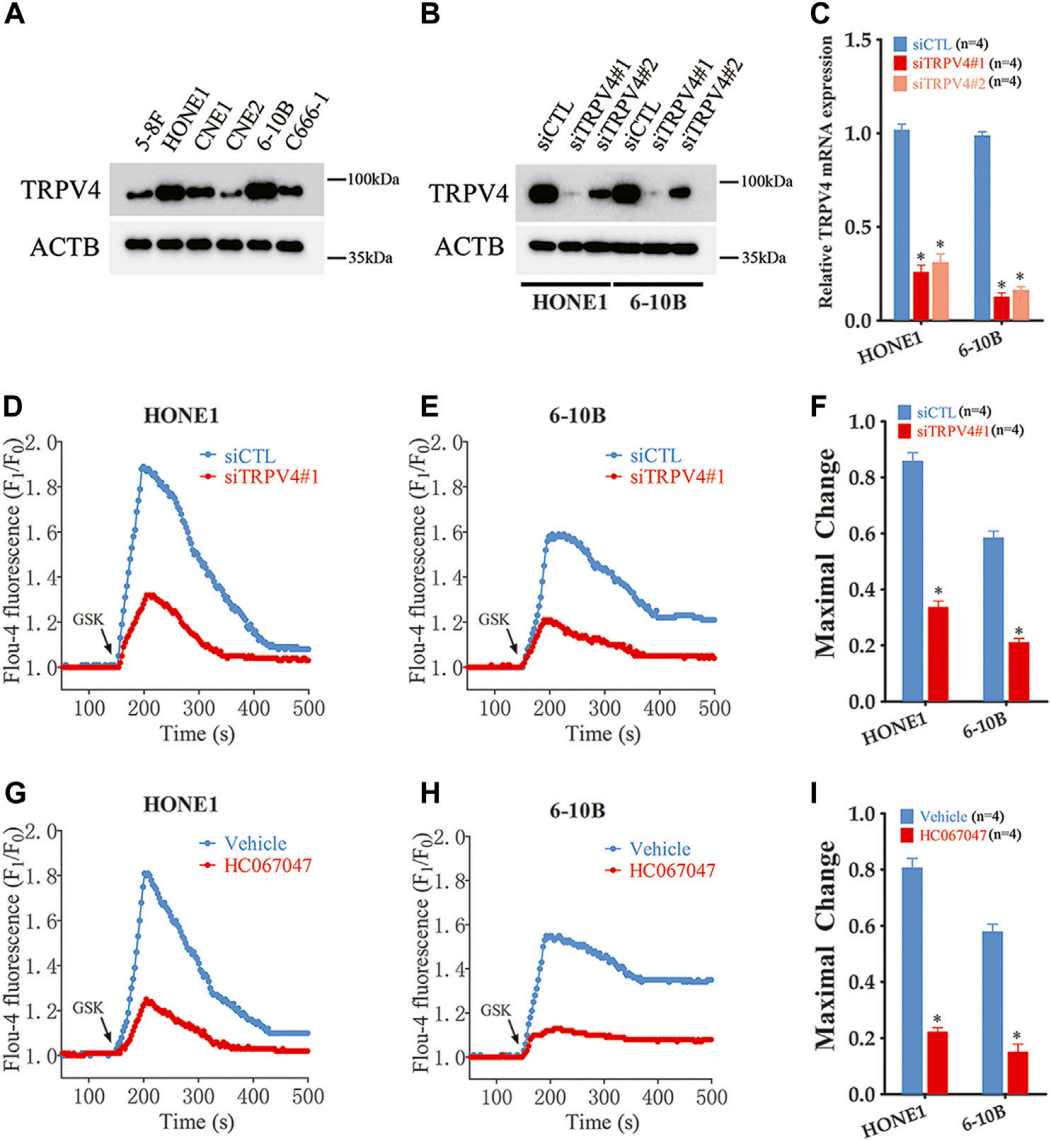
TRPV4 is functional expressed in NPC cells **(A)** Immunoblot showing TRPV4 expression in NPC cells (n = 4). **(B,C)** TRPV4 protein and mRNA expression in NPC cells transfected with control siRNA (siCTL) or TRPV4 siRNA (siTRPV4#1 and siTRPV4#2) (n = 4). **(D,E)** Representative single measurement for levels of intracellular Ca^2+^ in response to GSK1016790A (GSK, 100 nM) in NPC cells transfected with siCTL or siTRPV4#1. Fluo-4 fluorescence was excited at 488 nm and the signals relative to the starting signal (F1/F0) were calculated to quantify the cytosolic Ca^2+^ change. **(F)** Summary data of maximal change for D and E (n = 5). **(G,H)** Representative single measurement for levels of intracellular Ca^2+^ in response to GSK (100 nM) in NPC cells pre-treated with vehicle (0.1% DMSO) or HC067047 (4 μΜ) as in D and E. **(I)** Summary data of maximal change for G and H (n = 5). The data is the mean ± SEM. *p < 0.05, *versus* vehicle or siCTL.

To further investigate the effects of TRPV4 on NPC cells, we analyzed its expression levels in different NPC cell lines (5–8F, HONE1, CNE1, CNE2, 6–10B and C666-1). TRPV4 expression was higher in the HONE1 and 6–10B cells (Figure 2A). Therefore, we knocked down TRPV4 in both cell lines, and confirmed decreased levels of TRPV4 mRNA and protein compared to that in the control (transfected with scrambled siRNA) cells (Figures 2B, C). The Ca^2+^ influx in the suitably treated cells was measured by Fluo-4/AM staining. As shown in Figures 2D, E, the intracellular Ca^2+^ levels increased rapidly in the control cells after treatment with the specific TRPV4 activator GSK1016790A (100 nM). In contrast, a significant decrease in Ca^2+^ level was observed in the TRPV4-knockdown NPC cells (Figures 2D–F). Furthermore, the selective TRV4 inhibitor HC067047 (4 μM) abrogated GSK1016790A (100 nM)-induced Ca^2+^ elevation in the NPC cells (Figures 2H–J). Taken together, TRPV4 functions as a Ca^2+^-permeable channel in NPC cells.

### TRPV4 activation promoted NPC cell growth *in vitro* and *in vivo*

**FIGURE 3:**
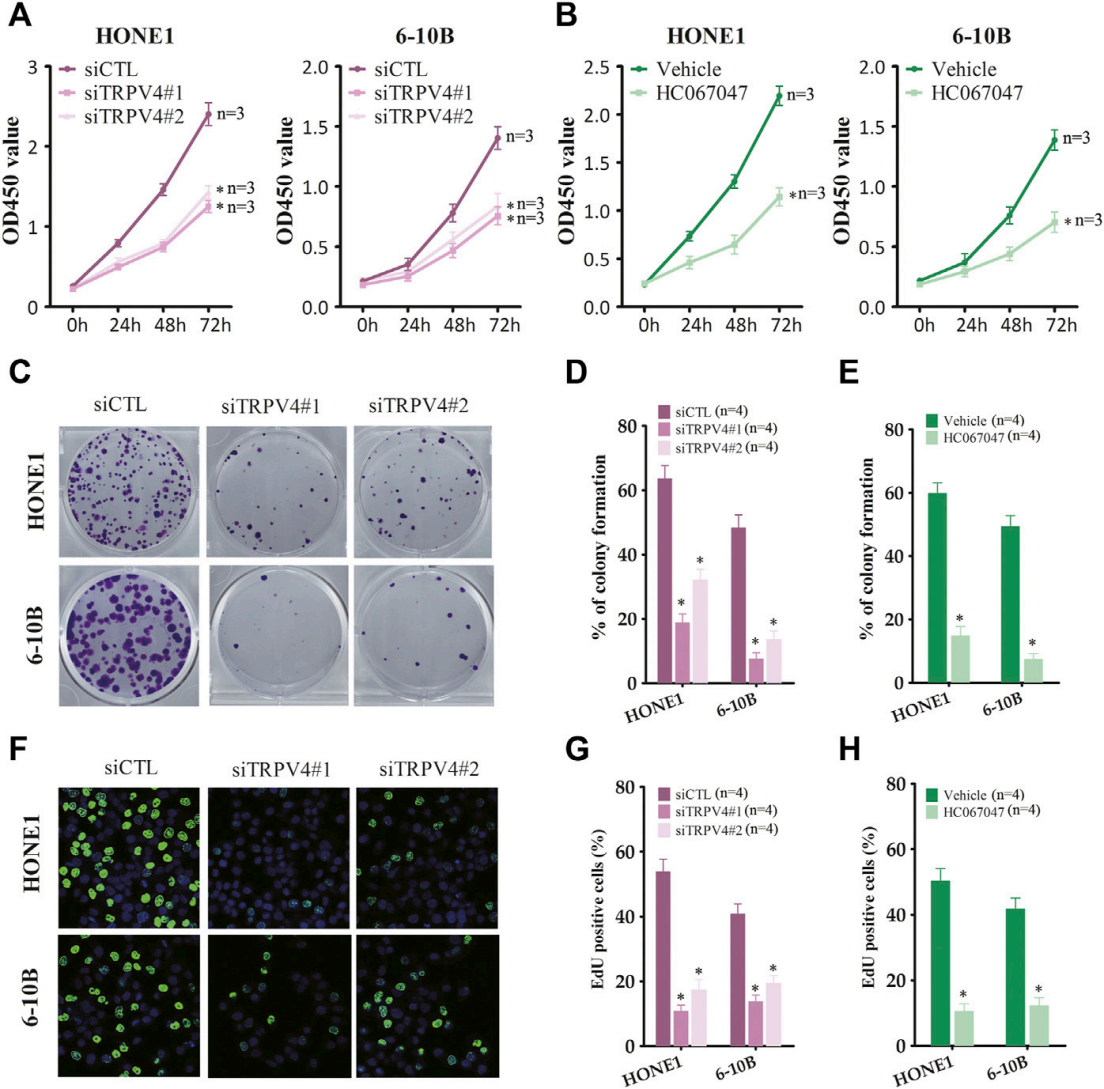
TRPV4 regulates NPC cells growth *in vitro* **(A)** Survival rates of HONE1 and 6–10B cells transfected with control siRNA (siCTL) or TRPV4 siRNA (siTRPV4#1 and siTRPV4#2) (n = 3). **(B)** Survival rates of HONE1 and 6–10B cells pre-treated with vehicle (0.1% DMSO) or HC067047 (4 μΜ) (n = 3). **(C,D)** Representative images and number of colonies formed by NPC cells transfected with siCTL, siTRPV4#1 or siTRPV4#2 (n = 4). **(E)** Number of colonies formed by NPC cells pre-treated with vehicle (0.1% DMSO) or HC067047 (4 μΜ) (n = 4). **(F,G)** Representative images and number of Edu-positive proliferative NPC cells transfected with siCTL, siTRPV4#1 or siTRPV4#2 (n = 4). **(H)** Edu-positive NPC cells pre-treated with vehicle (0.1% DMSO) or HC067047 (4 μΜ) (n = 4). The data represent the mean ± SEM. *p < 0.05, *versus* vehicle or siCTL.

**FIGURE 4:**
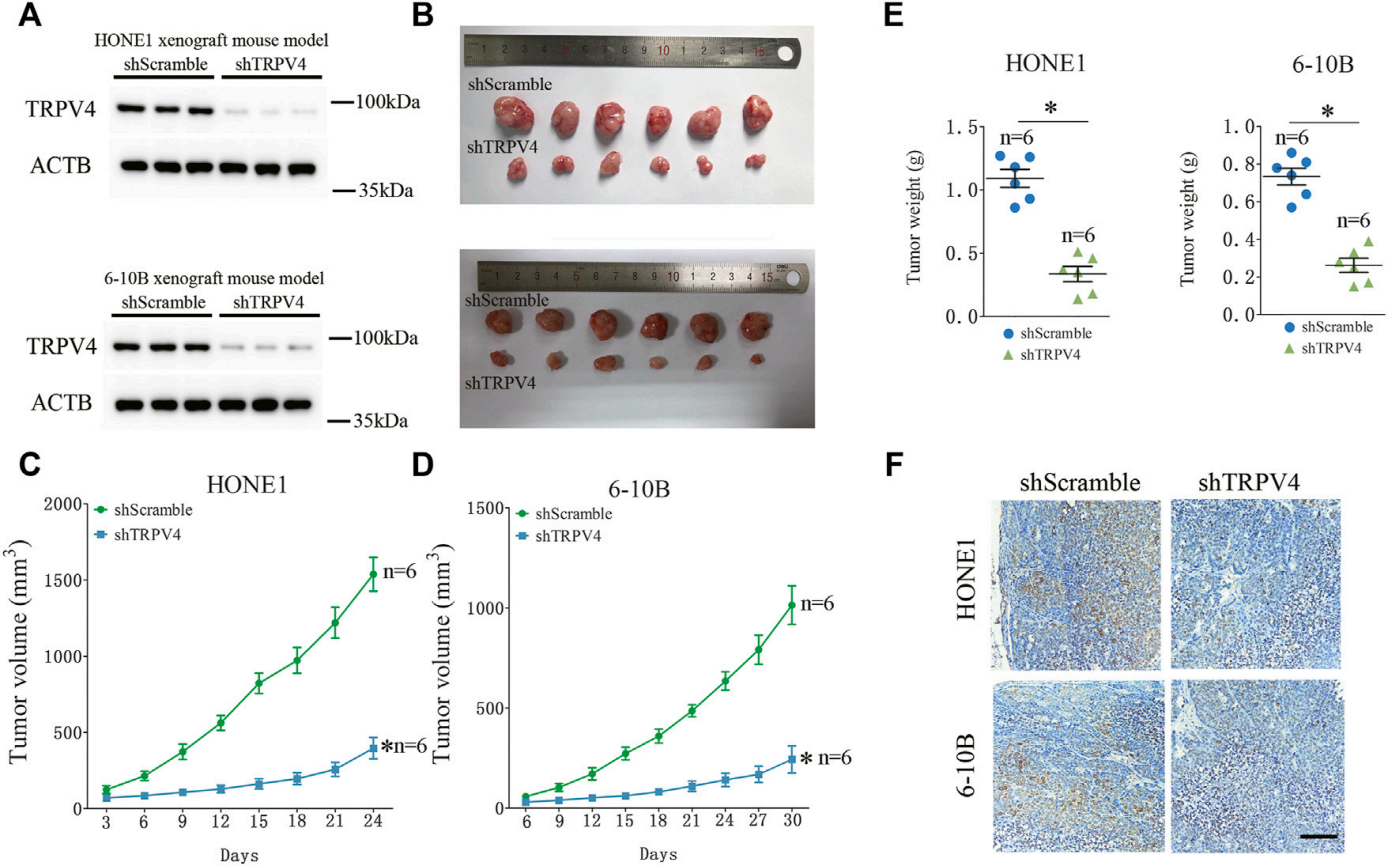
TRPV4 regulates NPC cells growth *in vivo* **(A)** Immunoblot showing TRPV4 expression in the xenografts of NPC cells transfected with shScramble or shTRPV4 (n = 3). **(B–D)** Representative images of tumors from the indicated groups (n = 6). **(E)** The tumor weight in the indicated groups (n = 6). **(F)** Representative images of immunohistochemical staining of Ki-67 in HONE1 (upper) or 6–10B (down) xenograft tumor tissues transfected with shScramble or shTRPV4 (n = 6). Scare bar = 100 μm. The data represent the mean ± SEM. *p < 0.05, *versus* shScramble.

To determine whether TRPV4 plays a key role in the growth of NPC cells, we first measured the viability of the TRPV4-knockdown HONE1 and 6–10B cells. As shown in Figure 3A, TRPV4 silencing decreased the viability rates of both cell lines. Likewise, inhibition of TRPV4 also decreased the growth of NPC cells (Figure 3B and Figure S1). Furthermore, TRPV4 blockade decreased the number of colonies formed by the NPC cells (Figures 3C–E). Consistent with the above results, the proportion of EdU-positive proliferating NPC cells reduced significantly following TRPV4 knockdown or HC0670476 (4 μM) treatment (Figures 3F–H). To validate the *in vitro* results, we established an *in vivo* xenograft model by subcutaneously injecting nude mice with control or TRPV4-knockdown HONE1 and 6–10B cells. TRPV4 expression was markedly decreased in tumor tissues from the TRPV4-knockdown group compared to the control group (Figure 4A). Furthermore, the tumors derived from NPC cell lines with TRPV4 knockdown were significantly smaller, both in terms of weight and volume, compared to those formed by the control cells (Figures 4B–E). The low *in situ* expression of Ki-67 in the TRPV4-knockdown tumor tissues also confirmed its suppressive effect on NPC cell growth (Figure 4F). Taken together, TRPV4 functions as an oncogene in NPC cells and promotes their growth *in vitro* and *in vivo*.

### TRPV4 regulates ca^2+^/calcineurin/NFAT4 axis to control NPC cell growth

**FIGURE 5:**
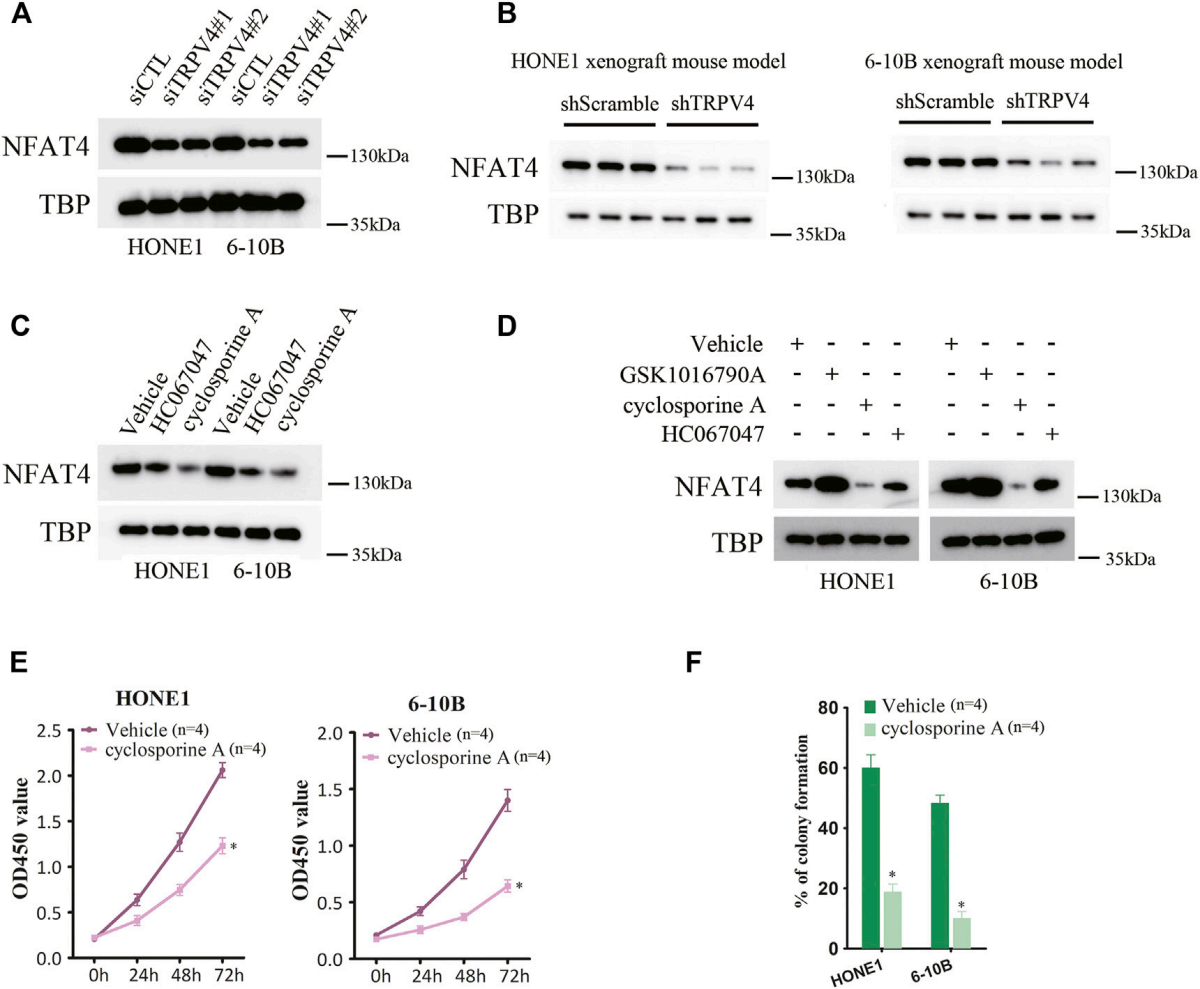
Inhibition of TRPV4/calcineurin suppresses NFAT4 activation **(A)** Immunoblot showing NFAT4 protein expression in the nuclear fraction of TRPV4-knockdown NPC cells (n = 3). **(B)** Immunoblot showing NFAT4 expression in the nuclear fraction of control or TRPV4-knockdown tumor cells from xenografts (n = 3). **(C)** Immunoblot showing NFAT4 expression in the nuclear fraction of NPC cells pretreated with HC067047 or cyclosporine A (n = 3). **(D)** Immunoblot showing NFAT4 expression in the nuclear fraction of NPC cells pretreated with HC067047 or cyclosporine A before exposure to GSK1016790A (n = 3). **(E,F)** The effect of cyclosporine A on **(E)** cell proliferation and **(F)** colony formation (n = 4). The data represent the mean ± SEM. *p < 0.05, *versus* shScramble. siCTL or Vehicle.

**FIGURE 6:**
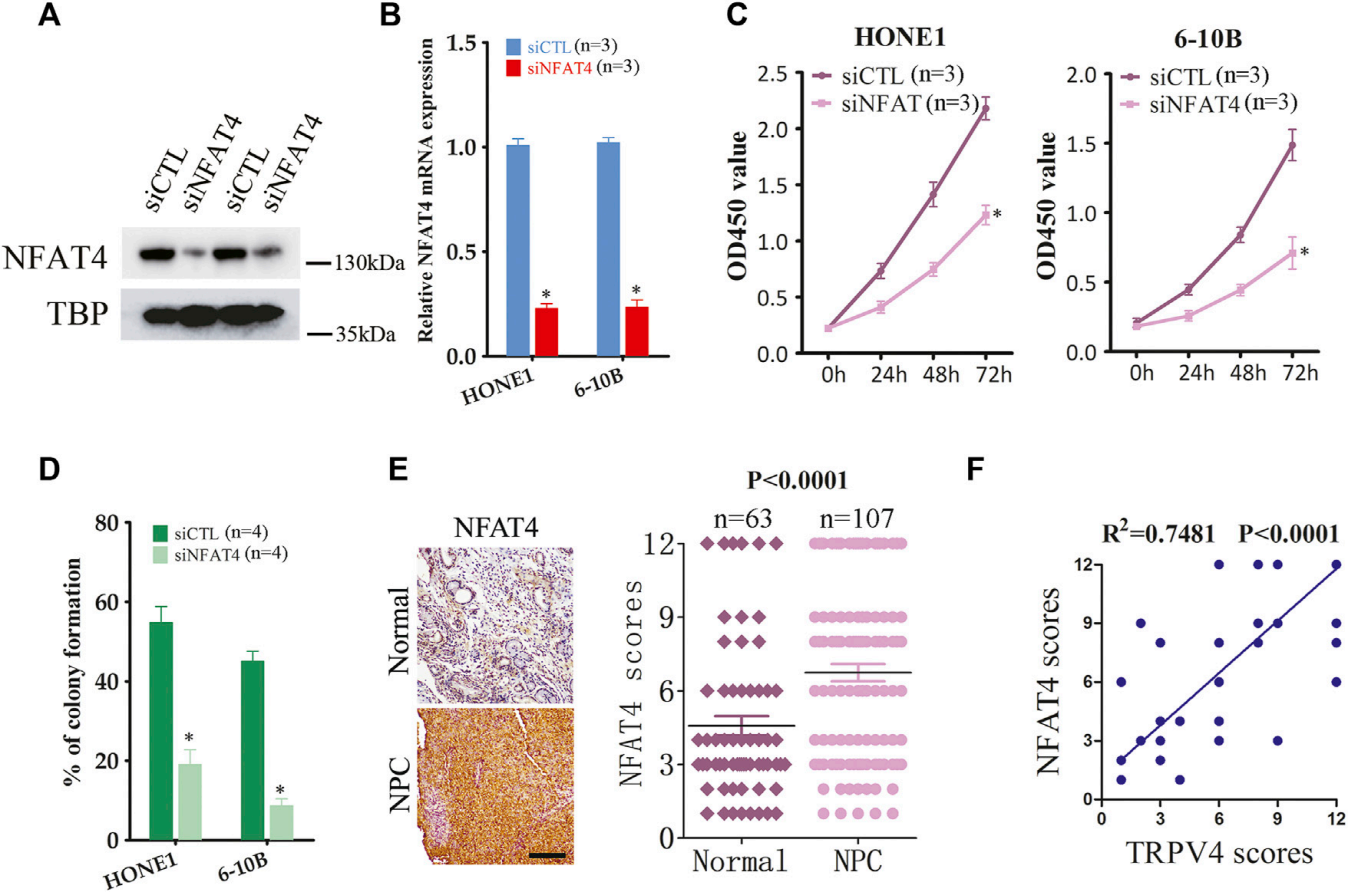
NFAT4 regulates the growth of NPC cells **(A,B)** NFAT4 protein and mRNA expression in NPC cells transfected with control siRNA (siCTL) or NFAT4 siRNA (siNFAT4) (n = 3). **(C)** Proliferation rates of the control and NFAT4-knockdown NPC cells (n = 3). **(D)** Number of colonies formed by the control and NFAT4-knockdown NPC cells (n = 4). **(E)** Representative images and scores of NFAT4 protein expression in normal (n = 63) and NPC tissues (n = 107). Scare bar = 100 μm. **(F)** Pearson correlation of NFAT4 and TRPV4 expression levels, *R* ^2^ = 0.7482 means that ∼75% variation in NFAT4 protein expression can be explained by TRPV4 protein expression, or the Pearson’s correlation between NFAT4 and TRPV4 is R = 0.8649. The data represent the mean ± SEM. *p < 0.05, *versus* siCTL.

To address the mechanisms underlying the oncogenic effects of TRPV4, we examined its downstream pathways in the NPC cells. Ca^2+^/calcineurin/NFAT signaling plays a key role in tumor progression. Moreover, TRPV4-regulated Ca^2+^ influx in ASMCs activates NFAT4 and promotes cellular proliferation. We found that silencing TRPV4 in the HONE1 and 6–10B cells decreased NFAT4 expression in the nucleus (Figure 5A). Likewise, TRPV4-knockdown tumors also expressed lower levels of NFAT4 in the nuclear fraction (Figure 5B). Consistent with these results, pharmacological inhibition of TRPV4 with HC067047 (4 μM) also reduced NFAT4 activation (Figure 5C). Moreover, inhibition of Ca^2+^-dependent calcineurin activation by cyclosporine A (CsA) markedly reduced accumulation of NFAT4 in the nucleus (Figure 5C). Notably, GSK1016790A (100 nM)-induced NFAT4 activation was suppressed by inhibiting calcineurin and TRPV4 (Figure 5D). CsA also inhibited growth and clonogenic ability of the NPC cells (Figures 5E,F). Next, we examined the effect of NFAT4 knockdown on the NPC cells using specific siRNA, and confirmed the decrease in NFAT4 mRNA and protein expression (Figures 6A,B). NFAT4 silencing markedly reduced cell viability compared to the control siRNA (Figure 6C), and decreased the number of colonies formed *in vitro* (Figure 6D). Collectively, these data suggest that TRPV4 drives NPC cell growth through the Ca^2+^/calcineurin/NFAT4 axis. NFAT4 protein expression was also significantly higher in the NPC tissues compared to that in the normal nasopharynx tissues (Figure 6E). The Pearson’s correlation between NFAT4 and TRPV4 is R = 0.8649, therefore NFAT4 protein expression was positively correlated with TRPV4 protein expression (Figure 6F).

## Discussion

We found that TRPV4 and NFAT4 were upregulated in NPC tissues and correlated positively with each other. Our study demonstrates for the first time that TRPV4 promotes NPC cell growth *via* the Ca^2+^/calcineurin/NFAT4 axis, and highlights the therapeutic potential of targeting the TRPV4/Ca^2+^/calcineurin/NFAT4 signaling pathway.

Studies increasingly show that the aberrant expression of TRPV4 in tumor tissues is associated with cancer progression (Nilius et al., 2007; Prevarskaya et al., 2007; Fiorio Pla et al., 2012; Xie et al., 2017; Zhang et al., 2018; Liu et al., 2021). However, the potential role of TRPV4 in the development and progression of NPC is still unclear. We found that both pharmacological inhibition as well as gene silencing of TRPV4 retarded the growth of NPC cells *in vitro*. Furthermore, the TRPV4-knockdown xenografts were smaller compared to the control tumors. Consistent with the present findings, we previously reported that knocking down TRPV4 suppressed colon cancer cell growth *via* PTEN activation, and inhibited invasiveness by blocking ZEB1 signaling (Liu et al., 2019; Zhang et al., 2021). Analysis of the data from The *Cancer* Genome Atlas and the Genotype-Tissue Expression platform also identified TRPV4 as an oncogene and a prognostic marker in colon cancer (Wang et al., 2021). Furthermore, pharmacological activation of TRPV4 is sufficient to trigger EMT of breast cancer cells (Azimi et al., 2020). TRPV4 is also overexpressed in hepatocellular carcinoma patients with high histological grade (Fang et al., 2018), and TRPV4-mediated Ca^2+^ entry enhanced gastric cancer cell proliferation, migration and invasion (Xie et al., 2017). In addition, TRPV4 expression was positively correlated with the size of non-small cell lung cancer tumors, and TRPV4 activation promoted proliferation of A549 cells (Pu et al., 2022). In contrast, low expression level of TRPV4 has been reported in the keratinocytes of non-melanoma skin cancer patients (Fusi et al., 2014), and TRPV4 activation in melanoma cells resulted in increased exocytosis and ferroptosis rates (Li et al., 2022). Activation of TRPV4 normalized tumor vasculature and improved cisplatin therapy from a transgenic adenocarcinoma mouse prostate model (Adapala et al., 2016). Consistent with this, cannabidiol-induced death of glioma cells is mediated *via* TRPV4-driven mitophagy (Huang et al., 2021). Therefore, TRPV4 is a double-edged sword in cancer, and its function depends on the tumor and cell type. TRPV4 is widely expressed in vasculature, fat tissues, liver, heart, lung, brain and many other organs, therefore the systemic use of antagonists of TRPV4 may induce adverse reactions (Kassmann et al., 2013). Thus, the exact role of TRPV4 in cancer and targeted drug delivery need to be investigated further. Ca^2+^ signaling plays key role in tumor progression (Roberts-Thomson et al., 2019). Consistent with the fact that TRPV4 is a Ca^2+^-permeable non-selective channel, the activation of TRPV4 by GSK1016790A increased Ca^2+^ influx in NPC cells, which was suppressed by pharmacological inhibition or gene silencing of TRPV4. We and others have found that TRPV4 regulates intracellular Ca^2+^ levels in colon cancer (Liu et al., 2019), gastric cancer (Xie et al., 2017), and hepatocellular carcinoma (Fang et al., 2018). Furthermore, the calcineurin-NFAT pathway is sensitive to Ca^2+^ fluctuation, and regulates transcription of target genes, including those involved in cancer progression. There is also evidence that activated Ca^2+^-calcineurin-NFAT4 signaling promotes stemness in oral squamous cell carcinoma cells (Lee S. H. et al., 2019), as well as cell migration and angiogenesis in breast cancer (Liu et al., 2018b) and colon cancer (Liu et al., 2018a). Moreover, Ca^2+^ influx through TRPV4 activates NFAT4 in ASMCs through calcineurin (Zhao et al., 2014). Pharmacological inhibition or gene silencing of TRPV4 reduced NFAT4 activation in NPC cells. Moreover, the expression of nuclear NFAT4 was also suppressed in the xenografts with TRPV4 down-regulation. Thus, we explored whether calcineurin-NFAT4 pathway lies downstream of TRPV4. The calcineurin inhibitors CsA and FK506 not only reduced NFAT4 accumulation in the nucleus, but also reversed TRPV4-induced NFAT4 activation. Furthermore, NFAT4 knockdown, and treatment with CsA and FK506 impaired the growth of NPC cells. Other studies have also supported the critical role of the NFAT pathway in cancer progression (Mancini and Toker 2009; Kim et al., 2019; Wu et al., 2021). Consistent with this, NFAT4 expression was higher in NPC tissues, and correlated positively with that of TRPV4. Therefore, our findings strongly suggest that TRPV4 exerts its oncogenic effects in NPC *via* the calcineurin-NFAT4 pathway.

In conclusion, TRPV4 stimulates NPC tumorigenesis by activating the Ca^2+^/calcineurin/NFAT4-signaling pathway, and the TRPV4/NFAT4 axis is potential therapeutic target for NPC.

## Data Availability

The original contributions presented in the study are included in the article/Supplementary Material, further inquiries can be directed to the corresponding authors.
