# Supplementary material for: Transient receptor potential vanilloid type 4 (TRPV4) promotes tumorigenesis via NFAT4 activation in nasopharyngeal carcinoma
Source: Front Mol Biosci. 2022 Dec 22;9:1064366. doi: 10.3389/fmolb.2022.1064366 (PMC9815116; doi:10.3389/fmolb.2022.1064366)
Supplement: Supplementary file 1 [file Table1.DOC]

**Supplementary Material**


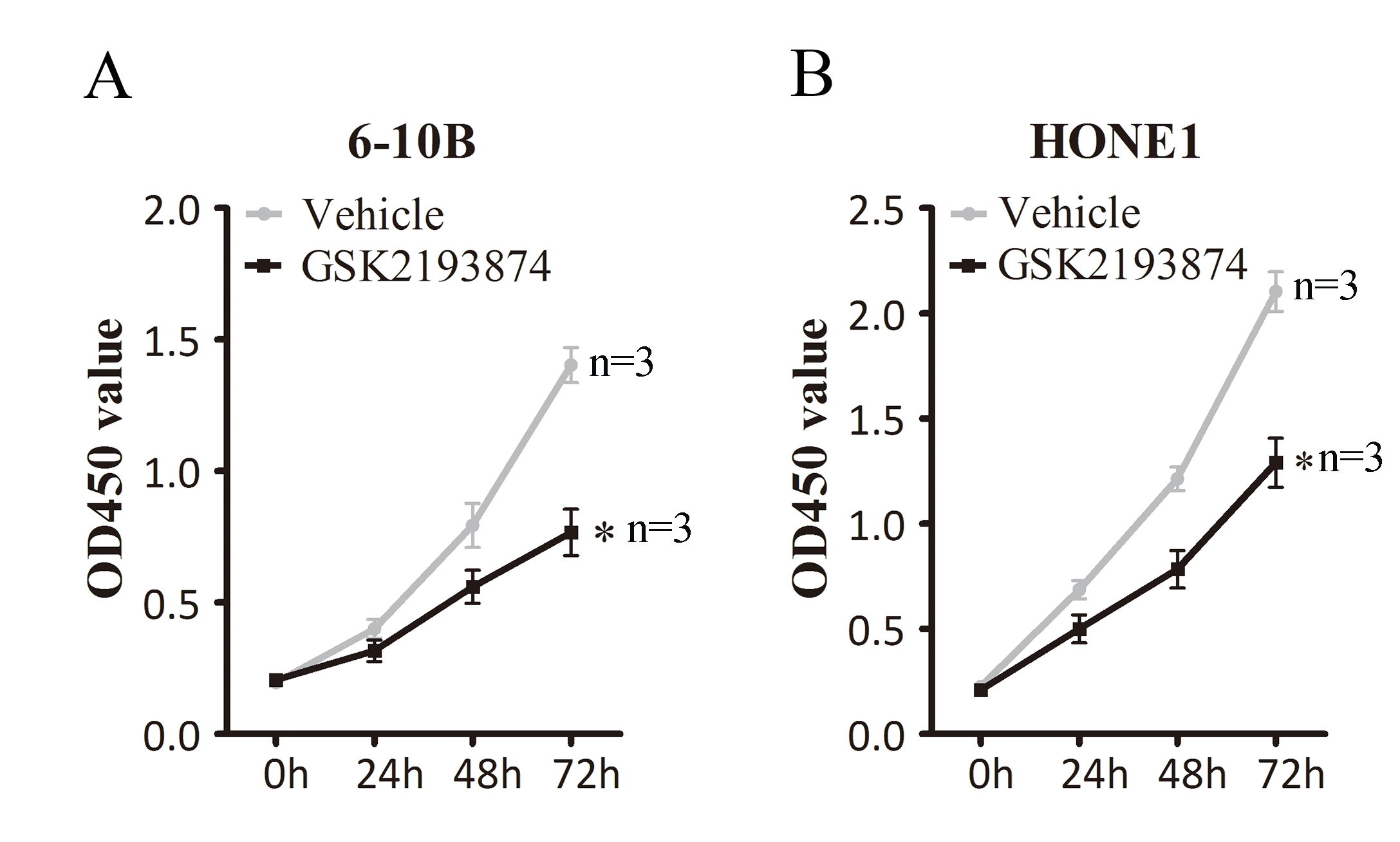


Supplementary Figure S1. Survival rates of 6-10B and HONE1 cells pre-treated with vehicle (0.1% DMSO) or GSK2193874 (5μΜ) (n=3). Values are means ± SEM. * p<0.05, versus vehicle

Table S1 Clinicpathological characteristics of NPC patients

|  | All Patients  (n=120) | |
| --- | --- | --- |
| Characteristic | n | % |
| Age(years) |  |  |
| ≤50 | 43 | 35.8 |
| >50 | 77 | 64.2 |
| Sex |  | |
| Male | 92 | 78.3 |
| Female | 28 | 21.7 |
| T stage |  |  |
| T1-T2 | 51 | 42.5 |
| T3-T4 | 69 | 57.5 |
| N stage |  |  |
| N0-1 | 74 | 61.7 |
| N2-3 | 46 | 38.3 |
| M stage |  |  |
| M0 | 53 | 44.2 |
| M1 | 67 | 55.8 |
| TNM stage |  |  |
| I-II | 75 | 62.5 |
| III-IV | 45 | 37.5 |
| Smoking |  |  |
| Yes | 82 | 68.3 |
| No | 38 | 31.7 |
